# Plasmodium TatD-Like DNase Antibodies Blocked Parasite Development in the Mosquito Gut

**DOI:** 10.3389/fmicb.2018.01023

**Published:** 2018-05-18

**Authors:** Wei Wang, Fei Liu, Ning Jiang, Huijun Lu, Na Yang, Ying Feng, Xiaoyu Sang, Yaming Cao, Qijun Chen

**Affiliations:** ^1^Key Laboratory of Zoonosis, Jilin University, Changchun, China; ^2^Department of Immunology, China Medical University, Shenyang, China; ^3^Key Laboratory of Zoonosis, Shenyang Agricultural University, Shenyang, China

**Keywords:** plasmodium, malaria, TAtD-like DNase, antibody, transmission blocking vaccine

## Abstract

The TatD-like DNase of *Plasmodium* species has previously been characterized as a conserved antigen that plays an important role in immune evasion. Here, we found that TatD-like DNase is expressed, apart from the erythrocytic stage, throughout the developmental stages of the parasite in the mosquito vector. Antibodies to the molecule significantly blocked parasites development and transition in the mosquito gut. Further, mice immunized with recombinant TatD-like DNase showed significant resistance to parasite challenge. The antigenicity of the TatD-like antigen in combination with various adjuvants, including Freund’s adjuvants, Montanide ISA 51 and 61, Alhydrogel (aluminum hydroxide), and levamisole was investigated. It was found that immunization of the recombinant TatD-like DNase in combination with Montanide ISA 51 induced strong **humoral** responses that showed significant protection against parasite challenge in a mouse model. The data further support that TatD-like DNase is a functionally important molecule in the whole development cycle of the malaria parasites and a candidate for malaria vaccine development.

## Introduction

The TatD DNase is a conserved protein present in most prokaryotic and eukaryotic species (Chen et al., 2014). We have recently identified a homologous protein in the *Plasmodium* spp., named TatD-like DNase, which was implicated as a parasite virulent factor (Chang et al., 2016). One of the functions of the *Plasmodium* TatD-like DNase was to counteract the extracellular traps (ETs) formed by the DNA and proteases released by macrophages and neutrophils during microbe infection (Biggs et al., 1991; Brinkmann et al., 2004; Yousefi et al., 2008; Urban et al., 2009; Marin-Esteban et al., 2012). Previous studies with immunofluorescent and immunoelectron microscopy approaches revealed that the TatD-like DNase is synthesized in the cytoplasm, translocated to the parasitophorous membrane and secreted outside the infected erythrocyte (Chang et al., 2016). The transcription and expression were associated with parasite virulence. Further, infectivity of the parasites was significantly attenuated with the deletion of the gene encoding the TatD-like DNase protein. Mice immunized with the recombinant TatD-like DNase protein combined with Freund’s adjuvant showed significant resistance to parasite infection.

In this study, to further demonstrate that the TatD-like DNase is a functionally critical molecule in the development and transmission of the malaria parasites, we investigated the expression of the molecule in the male and female gametocytes of *Plasmodium falciparum*, and on ookinetes and sporozoites in *Plasmodium berghei*. We found that the TatD-like DNase was expressed on the surface of gametocytes and ookinetes, but with lower expression in the sporozoites. Further, immunization with the recombinant TatD-like DNase in combination with Montanide ISA 51 generated specific antibodies that significantly inhibited parasite development.

## Materials and Methods

### Animals and Ethics Statement

The *P. berghei* ANKA strain was maintained in female BALB/c mice by serial mechanical passages, and used for challenge infection. Adult *Anopheles stephensi* (Hor strain) mosquitoes were maintained in 10% (w/v) glucose solution at 25°C and 50–80% relative humidity with a 12 h light/dark cycle in an insectary.

All laboratory animal protocols and procedures were performed following the regulations of the Animal Welfare and Research Ethics Committee of both Jilin University and China Medical University. Six- to eight-week-old BALB/c mice for the immunization and challenge experiments were purchased from Experimental Animal Center of Jilin University (Changchun, China).

### Sequence Analysis of the TatD-Like DNase Genes in *P. falciparum* Isolates

DNA samples were purified from *P. falciparum* 3D7, FCR3S 1.2, and AH1 clones after *in vitro* cultivation. Eighteen DNA samples originally purified from isolates of patients in the Friendship Hospital of the Peking Union Medical College were also included in the study. The following primers were designed based on the genomic sequence of the TatD-like DNase gene (PF3D7_0112000 in the PlasmoDB database) of the *P. falciparum* 3D7 clone and were used for amplification of the TatD-like DNase gene: forward primer, 5′-AAA TTA GTT TTT CAT TAT ATT AAA TAT ATA-3′; reverse primer, 5′-ACC TCA GTT TCT TGA ACA AAT TC-3′. Amplification parameters were: 94°C for 5 min and 35 cycles of 94°C for 30 s, 50°C for 30 s, and 60°C for 30 s. PCR products were cloned into pMD18-T vector (Takara, Dalian, China) and sequenced. The sequence from each parasite isolate was analyzed using the software DNAMAN 7 (Lynnon Biosoft).

### Preparation of Recombinant TatD-Like DNase of *P. berghei*

The genes encoding the His- and GST-tagged TatD-like DNase of *P. berghei* (PBANKA_0201800) were cloned into the pET-28a and pGEX-4T-1 vectors, respectively (Invitrogen), and expressed in *Escherichia coli* BL21(DE3), as described in our earlier study (Chang et al., 2016). His- and GST-tagged recombinant proteins were purified using the His GraviTrap^TM^ system (GE Healthcare) and the Glutathione Sepharose^TM^ 4B system (GE Healthcare), respectively, according to the manufacturer’s instructions. Purified proteins were analyzed with SDS-PAGE and Western blots before further experiments.

### Preparation of *P. falciparum* Gametocytes

The enrichment of gametocytes of *P. falciparum* was carried out as previously described (Delves et al., 2016). The asexual stage of the *P. falciparum* 3D7 strain was synchronized on day one with 2–3% parasitemia in 3% hematocrit. Half of the spent medium was changed on day 2 when the parasites were in ring stage. The stressed schizont stage parasite was separated at 2–3% parasitemia in 5% hematocrit with spent medium. Heparin was added on day 4 at a concentration of 20 U/ml for 4 days to eliminate parasites on the asexual stage. Gametocytes at stages I, II, III, IV, and V were harvested on days 4, 6, 8, and 14, respectively.

### Expression Analysis of TatD-Like DNase on *P. falciparum* Gametocytes

Thin smears of the infected erythrocytes at different stages were fixed with pre-cooled methanol at -80°C for 15 min, followed by incubation in PBS containing 0.1% Triton X-100 for 15 min, and then washed three times with PBS at room temperature for 5 min. The slides were blocked in 5% defatted milk in PBS for 1 h, then incubated with a rabbit anti-PfTatD-like DNase serum (1:100) and a control serum overnight at 4°C. The smears were incubated with Alexa Fluor 488 conjugated goat anti-rabbit IgG (Life Technologies, CA, United States; 1:1,000) for 1 h at 37°C. After three washes with sterile PBS, the nuclei of the parasite were stained with 1 μg/ml of 4′,6-diamidino-2-phenylindole (DAPI) for 5 min at room temperature. Fluorescent images were captured with a fluorescence microscope (Olympus, BX 53, Japan).

### Expression Analysis of the TatD-Like DNase on Parasites in the Mosquito Stages

*Plasmodium berghei* schizonts, gametocytes, and subsequent developmental stages (ookinetes and sporozoites) were washed once in PBS and then fixed with 4% paraformaldehyde (Sigma, CA, United States) and 0.0075% glutaraldehyde (Sigma) in PBS for 20 min at room temperature. After washing in PBS, parasites were rinsed with 50 mM glycine in PBS and blocked with PBS containing 5% defatted milk for 1 h at 37°C. After three subsequent washes in PBS, the slides were incubated with an anti-TatD-like DNase serum (1:500 dilution) diluted in 5% defatted milk at 37°C for 1 h and then incubated with FITC-labeled goat anti-rabbit IgG (1:500, Invitrogen, CA, United States) at 37°C for 1 h. The parasite nuclei were counterstained with 1 μg/ml DAPI. ProLong^®^ Gold Antifade Reagent (Invitrogen) was added to the samples and images were captured with an Olympus BX53 fluorescence microscope (Olympus Corporation).

### Immunogenicity Analysis of the *Plasmodium* TatD-Like DNase in Combination With Different Adjuvants

BALB/c mice were randomly assigned into 12 groups, with eight mice in each group. **Six** groups were immunized with His-tagged *P. berghei* (Pb)TatD-like DNase formulated with five adjuvants including Montanide ISA51, 61 (SEPPIC, Paris, France), levamisole (Sigma), Freund’s adjuvant (Sigma), Alhydrogel (aluminum hydroxide wet gel, Invivogen, United States), and PBS. The other 6 groups were immunized with GST protein formulated with the same adjuvants as negative controls. Mice were intramuscularly immunized with the antigen-adjuvant emulsion at weeks 0, 2, 4, and 6. Each mouse was immunized with 25 μg of antigen emulsified with an equal volume of the adjuvant. Blood samples were collected from mice before the first and two days before each immunization. Sera were collected from the blood samples by centrifugation at 1,500 rpm for 10 min and stored at -80°C for further assays.

### Detection of Antibody Responses by Indirect Enzyme Linked Immunosorbent Assays

The titer of the immunized mice was measured by indirect enzyme linked immunosorbent assay (ELISA). ELISA plates (Nunc, Rochester, NY, United States) were coated with 50 μl of GST-tagged recombinant proteins per well diluted to a concentration of 5 μg/ml in coating buffer (15 mM Na_2_CO_3_, 35 mM NaHCO_3_, pH 9.6) at 4°C overnight. Plates were washed four times with washing buffer (PBS containing 0.05% Tween 20) and blocked with 3% bovine serum albumin (BSA) in PBS for 1 h at 37°C. After washing, 50 μl of serum samples at different dilutions (1:1,000, 1:2,000, 1:4,000, 1:8,000, 1:16,000, and 1:32,000) were added in triplicates to the wells and incubated for 1 h at 37°C. Plates were then washed four times and incubated with alkaline phosphatase-conjugated goat anti-mouse IgG (1:20,000 dilution, Sigma, St Louis, United States) for 1 h at 37°C. After washing, 50 μl of substrate solution containing 4-nitrophenyl phosphate disodium salt hexahydrate (Sigma) and 9.7% diethanolamine (pH 9.8) was added to each well to detect the antigen-antibody reaction. After incubation for 15 min, the optical density of each well was read at 405 nm in a Biotek micro-ELISA auto-reader 808 (BioTeK Instruments, Winooski, VT, United States). The experiment was repeated three times and the OD_405_ values represented by the mean ± SD.

### Immune Protection Assay

Each immunized mouse was challenged by intraperitoneal injection of 1 × 10^6^ *P. berghei* ANKA strain-infected erythrocytes (iRBCs) after the specific antibody titer reached 1:32,000. Afterwards, thin blood smears were prepared every day and stained with Giemsa solution (Sigma) to record the parasitemia and survival rate of the immunized mice.

### *In Vitro* Transmission Blocking Assay With TatD-Like DNase Specific Antibodies

To determine whether the **PbTatD-like DNase** specific antibodies could inhibit the exflagellation of male gametocytes, mice were treated with phenylhydrazine and intraperitoneally inoculated with 200 μl of iRBCs containing 1 × 10^6^ *P. berghei* parasites. Three days after infection, 10 μl of mouse blood was mixed with the ookinete culture medium containing an **anti-PbTatD-like serum** or **a control serum from GST-immunized mice** at 1:5, 1:10, and 1:50 dilutions. Quantification of exflagellation was performed. Briefly, 10 μl of gametocyte-infected blood was obtained from the tail vein and mixed immediately with 90 μl of complete ookinete culture medium. The mixture was placed under a Vaseline-coated coverslip at 25°C, and 15 min later exflagellation centers were counted over the next 10 min under a phase contrast microscope.

For the *in vitro* ookinete conversion assay, blood was collected from mice three days after infection. After removal of the serum, blood was reconstituted using the anti-PbTatD-like DNase serum or the control serum from GST-immunized mice at 1:5, 1:10, and 1:50 dilutions with the complete ookinete culture medium. Parasites were cultured *in vitro* at 19°C for 24 h. The cultures were fixed, labeled with the anti-Pbs21 mAb (1:500), and ookinete numbers quantified under a fluorescence microscope (Olympus BX53, Olympus Corporation).

### *In Vivo* Transmission Blocking Assay With TatD-Like DNase Specific Antibodies

To test the inhibitory effect of the TatD-like DNase specific antibodies on gametocyte exflagellation, each mouse (six per group) was intraperitoneally injected with 500 μl anti-TatD-like DNase serum or control serum for three days beginning on day 0 of *P. berghei* infection (1 × 10^6^ iRBCs per mouse). The gametocytemia was monitored at day three after infection, and the male gametocyte exflagellation was quantified as described above.

To test the inhibitory effect of the TatD-like DNase specific antibodies on oocyst formation, mice (six per group) were infected with 5 × 10^6^ *P. berghei* iRBCs as above. Each mouse was injected with 500 μl TatD-like DNase specific serum or control serum at day three after infection. One hour later, mice **were used to feed** with starved, 4-day-old female *A. stephensi* mosquitoes for 30 min. After removal of the unfed mosquitoes, the engorged mosquitoes were maintained in an insectary at 19–21°C and 70% relative humidity. Ten days after feeding, at least 50 mosquitoes were dissected from each group to determine the intensity of oocysts per midgut **under a microscope.**

GraphPad Prism 6.01 (GraphPad software, San Diego, United States), SPSS 19.0, and one-factor analysis of variance (ANOVA) were used in this study. Mean ± standard deviation (*SD*) was used to express the data. *P* values < 0.05 were considered significant between groups. Gametocytemia and ookinete conversion rates were analyzed by the Student’s *t*-test. The intensity of infection (oocysts/midgut) was analyzed by the Mann–Whitney *U*-test. *P*-values less than 0.05 were considered statistically significant.

## Results

### The Gene of TatD-Like DNase Is Conserved in *P. falciparum*

The genes encoding the TatD-like DNase **protein** of *P. falciparum* 3D7, AH1, FCR 3S 1.2 strains, and 15 wild isolates were amplified and sequenced. The coding regions of the genes are identical, and there are around 5% differences in the non-coding regions among the genes (**Supplementary Figure S1**).

### TatD-Like DNase Is Expressed on Male and Female Gametocytes and on Ookinetes in *Plasmodium* Parasites

**FIGURE 1:**
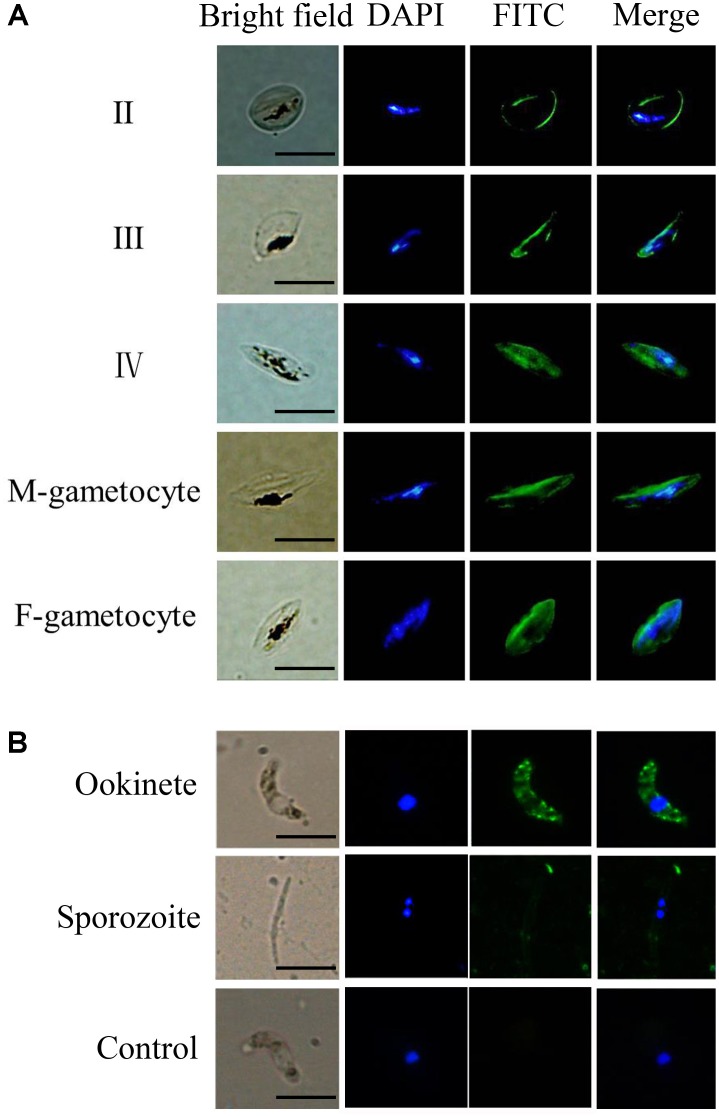
The TatD-like DNase is expressed during the sexual stages of the *Plasmodium* parasites. Blood smears containing gametocytes of *P. falciparum* parasites **(A)** and purified ookinetes and sporozoites *P. berghei* **(B)** were fixed and the expression of TatD-like DNase detected with anti-TatD-like DNase and FITC-conjugated goat anti-rabbit IgG (green) antibodies. Nuclei were stained by DAPI (blue). Naïve serum served as negative control. The expression of TatD-like DNase was detected on the surface of gametocytes, ookinetes, but not on the sporozoites. The negative control sera did not react with the parasite. Scale bar: 10 μm.

***Plasmodium falciparum* gametocytes** were obtained as previously described (Miao et al., 2013). ***P. berghei* ookinetes** were obtained by manual dissection of infected mosquitos. The expression of the TatD-like DNase was investigated by immunofluorescent assays, as described (Lal et al., 2009; Chang et al., 2016). Results clearly showed that the molecule was expressed on the surface of both male and female gametocytes and on ookinetes (**Figures 1A,B**).

### TatD-Like DNase Specific Antibodies Blocked Ookinete Conversion and Oocyst Formation Inside the Mosquito Vectors

**FIGURE 2:**
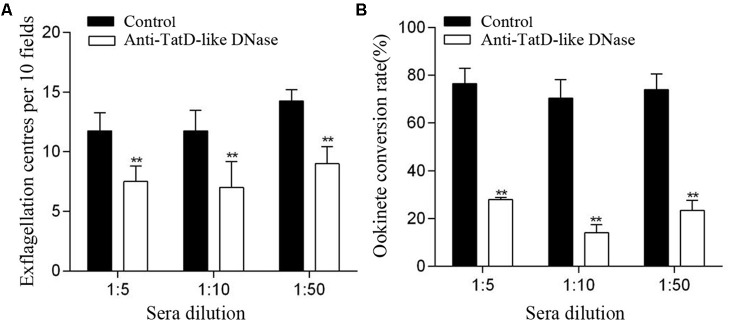
Anti-TatD-like DNase antibodies inhibited male gametocyte exflagellation and ookinete conversion *in vitro*. **(A)** Effect of anti-TatD-like antibodies on exflagellation of male gametocytes. Exflagellation centers per 10 fields of view were measured after 15 min incubation with an immune serum or a control serum at final dilutions of 1:5, 1:10 and 1:50. Means were calculated from three separate experiments. Error bars indicate mean ± SD (*n* = 3). ^∗∗^*P* < 0.01 (Student’s *t*-test). **(B)** Effect of anti-TatD-like antibodies at 1:5, 1:10, and 1:50 dilutions on *P. berghei* ookinete conversion *in vitro*. Mean values were calculated from three separate experiments. Error bars indicate mean ± SD (*n* = 3). ^∗∗^*P* < 0.01 (Student’s *t-*test).

**FIGURE 3:**
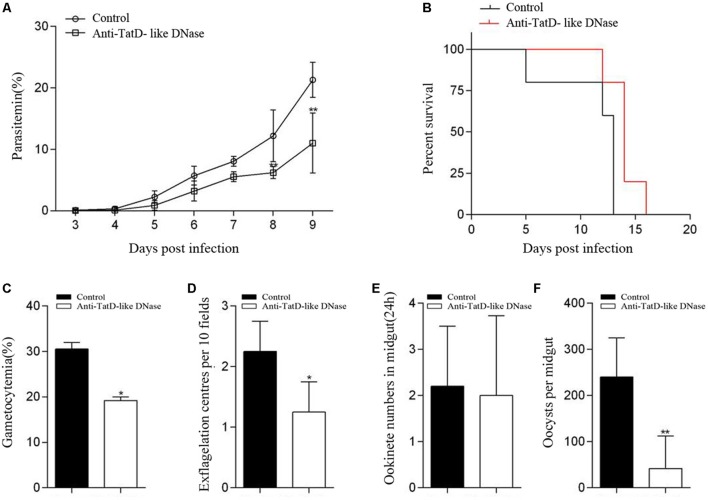
TatD-like DNase immune sera passively transferred in mice inhibited male gametocyte exflagellation, ookinete conversion and oocyst formation. Mice passively transferred with TatD-like DNase immune sera exhibited lower parasitemia **(A)** and survived longer than those of the control group **(B)**. The gametocytemia **(C)**, male gametocyte exflagellation **(D)**, midgut ookinete numbers **(E)** and oocyst numbers **(F)** were significantly lower in the group injected with immune sera than that of the control group. Error bars indicate mean ± SD (*n* = 3). ^∗^*P* < 0.05, ^∗∗^*P* < 0.01.

The potential transmission blocking effect of the TatD-like DNase specific antibodies was investigated using both *in vitro* and *in vivo* assays. After incubation with 1:5, 1:10, and 1:50 dilutions of anti-PbTatD-like DNase serum, the exflagellation of male gametocytes was significantly reduced by 36.2, 40.4, and 36.8%, respectively, as compared to the control sera (**Figure 2A**). Further, in the *in vitro* assay, the ookinete conversion rates were significantly reduced by 63.4, 70.3, and 68.4% for the 1:5, 1:10, and 1:50 dilutions, respectively, when treated by the immune sera, as compared to the control sera (**Figure 2B**). To examine the transmission blocking effect of the TatD-like DNase specific antibodies, naïve mice were passively transferred with the immune sera. After infection, a reduction in parasitemia (**Figure 3A**) and delayed death (**Figure 3B**) were observed in the TatD-like DNase antiserum group compared with the control groups. A reduction in gametocytemia (**Figure 3C**) and exflagellation (**Figure 3D**) by 37.2% and 44.4%, respectively, was observed. After direct feeding of mosquitoes, the midguts were dissected at 24 h and day 10. The ookinetes and midgut oocysts were counted. Compared to the control groups, the TatD-like DNase antiserum group showed no noticeable reduction in midgut ookinete numbers (**Figure 3E**) but an 82.5% reduction in midgut oocysts numbers (**Figure 3F**).

### Immunization of the Recombinant TatD-Like DNase in Combination With Montanide ISA 51 Generated Significant Protection Against Parasite Challenge

**FIGURE 4:**
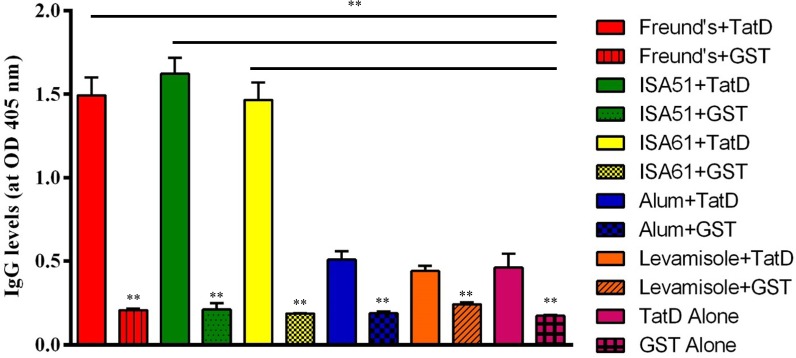
TatD-like DNase-specific IgG level response after immunization. BALB/c mice were immunized with TatD-like DNase of *P. berghei* in combination with Freund’s adjuvant (red), Montanide ISA 51 (green), Montanide ISA 61 (yellow), Alhydrogel (blue), Levamisole (orange), or with protein alone (pink). Antigen-specific IgG responses were detected by ELISA with sera diluted 1:16,000. Data is presented as mean ± SD. ^∗^*P* < 0.05, ^∗∗^*P* < 0.01.

His- and GST-tagged TatD-like DNase of *P. berghei* were expressed in *E. coli* and purified with the GraviTrap^TM^ and Glutathione Sepharose^TM^ 4B systems (**Supplementary Figure S2**). Antibody responses after immunization with the His-tagged recombinant proteins combined with five adjuvants were monitored with ELISA assays. The groups immunized with TatD-like DNase of *P. berghei* in combination with Montanide ISA 51 and 61, and Freund’s adjuvant showed significantly higher levels of antibody responses after four immunizations than the other groups. The groups immunized with the antigen in combination with Alhydrogel or levamisole did not show significantly different antibody responses than the control group (*P* < 0.01; **Figure 4**).

**FIGURE 5:**
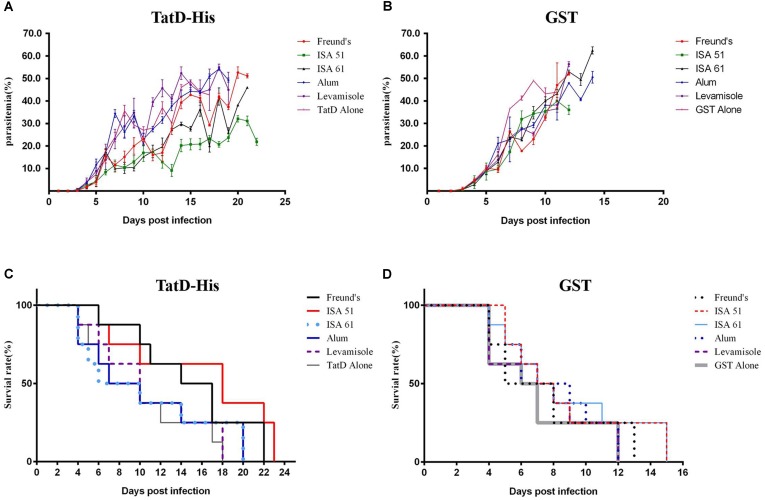
Protection of TatD-like DNase specific antibodies against parasite infection. One week after the last immunization, mice were intraperitoneally challenged with 1 × 10^6^ iRBC of *P. berghei*, and mice’s parasitemia **(A,B)** and survival rates **(C,D)** were recorded. Mice immunized with the antigen in combination with Montanide ISA 51 showed significantly higher protection than those in the other immunized groups.

One week after the last immunization, mice were challenged with 1 × 10^6^ *P. berghei* ANKA strain-infected iRBCs to evaluate the effect of the immune protection. Groups immunized with TatD-like DNase showed significantly lower parasitemia than the groups immunized with GST control protein (**Figures 5A,B**). Mice immunized with Montanide ISA 51, Montanide ISA 61, and Freund’s adjuvant exhibited significantly lower parasitemia than other groups, and the group immunized with Montanide ISA 51 showed a better control of parasitemia than groups immunized with Montanide ISA 61 or Freund’s adjuvant (**Figure 5A**). All mice immunized with GST control protein were dead within 15 days after challenge (**Figure 5D**). The groups immunized with the PbTatD-like DNase in combination with Montanide ISA 51 survived much longer than any other group (**Figures 5C,D**). The data revealed that Montanide ISA 51 plays an important role as adjuvant in vaccination against malaria.

## Discussion

The life cycle of *Plasmodium* parasites consists of sexual and asexual development stages in two hosts, human beings and anopheles mosquitoes. An efficient mechanism that counteracts the host immune response will provide essential protection for the successful development and transmission of the parasites. It is known that innate immune responses mediated by macrophages, NK cells, and oxygen radicals can sufficiently inhibit parasite development (Hou et al., 2016). We have previously reported that *Plasmodium* parasites exploit the TatD-like DNase protein to counteract host cellular responses in the asexual stages in human circulation, and that TatD-like DNase specific antibodies inhibit parasite proliferation in the blood (Chang et al., 2016). To further investigate the function of the TatD-like DNase protein in parasite development, we analyzed the expression and distribution of the TatD-like DNAse in the sexual development stages. Immune fluorescence assays with TatD-like DNAse specific antibodies indicated that TatD-like DNase is expressed on the surface of male and female gametocyte in *P. falciparum* and on *P. berghei* during the entire sexual stage (**Figures 1A,B**), raising the question of whether TatD-like DNase participates in immune evasion and promote fertilization in sexual reproduction. Inside the host, some of the merozoites after schizogony developed into gametocytes. The male gametocyte exflagellated and fertilized macrogametes to form zygotes, then sequentially transformed to ookinete, oocyst, and sporozoite before being transmitted to the human hosts (Bechtsi and Waters, 2017). Though we still cannot conclude the function of the TatD-like DNase in the sexual stages of the parasites, it is speculated that the molecule might protect the parasite from the oxidation stress in the mosquito gut. Earlier studies in comparing the genetically selected refractory and susceptible strains of *Anopheles gambiae* found that the refractory or resistant strain was in a chronic state of oxidative stress, which resulted in increased steady-state levels of reactive oxygen species and favor parasite melanization (Kumar et al., 2003). Our preliminary studies indicated that TatD-like DNase could protect the parasite from oxidation by facilitating the DNA repairing process (data not shown). Thus, the TatD-like DNase is likely a critical molecule for the successful development of the parasites inside the mosquitoes. The identification of TatD-like DNase on gametocytes, ookinetes, and oocysts indeed support the speculation.

We further investigated whether the TatD-like DNase specific antibodies could inhibit parasite development inside the mosquitoes. In both *in vitro* and *in vivo* assays, it was clearly observed that the male gametocyte exflagellation and macrogamete fertilization were significantly inhibited, and that the numbers of ookinetes and oocysts inside the mosquitoes were significantly reduced compared with the control groups (**Figures 3D–F**). Thus, the specific antibodies could inhibit parasite development in both the asexual and sexual stages. The data is of great importance in the development of a vaccine potent against the disease and in blocking transmission.

We then investigated the immunogenicity of the TatD-like DNase protein in combination with various adjuvants, including Montanide ISA 51 and 61, Alhydrogel, and levamisole. Previous studies indicated that Montanide ISA 51 is a strong Th-2 biased inducer (Yazdani et al., 2004; Parzych et al., 2017). Here we found that mice immunized with Montanide ISA 51 as an adjuvant generated a higher level of antibody responses than those in the other groups (P < 0.01, **Figure 4**), and the mice showed significantly stronger protection than the mice immunized with the same antigen combined with other adjuvants (**Figure 5**). Previous exploration on the malaria vaccine has been mainly carried out in two ways; one is single antigen vaccines, which facilitate the immune protection by immunization with single antigen plus an adjuvant. The most prominent vaccine is the RTS’S/AS vaccine, which is based on the repeated region of the circumsporozoite surface antigen (CSP). The idea was to block parasite invasion in hepatocytes and establishment in human hosts with anti-CSP antibodies (Agnandji et al., 2015). Additionally, several antigens such as merozoite surface antigens from the asexual stage parasite have also been tested to inhibit parasite proliferation in human erythrocytes, but the clinical protection effect has been limited (Matuschewski, 2017). The other approach was to block parasite development in the mosquito midgut by antibodies to the gametocytes and ookinetes (Parzych et al., 2017). Further, immunization with mixed or arrays of multistage antigens has also been tried, but the protection has not been satisfactory (Lyke, 2017; Matuschewski, 2017). The finding of an antigen expressed in both blood and sexual stages of the parasite will hopefully open a new avenue for malaria vaccine studies.

In conclusion, TatD-like DNase is a protein actively expressed on gametocyte and ookinete in the sexual development stages of *Plasmodium* parasites. Specific antibodies to the molecule inhibited exflagellation of male gametocyte and ookinete conversion in the midgut of mosquitoes. Immunization with recombinant TatD-like DNase in combination with Montanide ISA 51 generated significant protection after parasite infection. TatD-like DNase is a potential candidate for development of a vaccine for both transmission blocking and disease protection.

## Author Contributions

WW, FL, and NJ performed the main experiments. HL, NY, YF, and XS provided laboratory assistance. YC provided the mosquito facility and designed the experiment. QC designed and supervised the study and wrote the manuscript.

## Conflict of Interest Statement

The authors declare that the research was conducted in the absence of any commercial or financial relationships that could be construed as a potential conflict of interest.

## Supplementary Material

FIGURE S1 Sequence alignment analysis of the genes encoding TatD-like DNase in various *P. falciparum* strains and wild isolates. Numbers 1 to 15 represent the sequences amplified from wild type strains. PF_3D7, PF_AH1, PF_FCR3S represent the genomic sequences of the corresponding strain. PF_3D7_mRNA represents the mRNA sequence the *P. falciparum* 3D7 strain. The protein coding regions (dark blue) are completely conserved, whereas the differences only occurred in intron domains (white and pink).

FIGURE S2 Purification and confirmation of His- and GST-tagged recombinant proteins. Quality of the purified GST-tagged TatD-like DNase **(A)**, His-tagged TatD-like DNase **(B)**. TatD-like DNase was determined by Coomassie stained SDS-PAGE gels and Western blots with tag-specific antibodies.
