# Supplementary figures and images for: Plasmodium TatD-Like DNase Antibodies Blocked Parasite Development in the Mosquito Gut
Source: Front Microbiol. 2018 May 18;9:1023. doi: 10.3389/fmicb.2018.01023 (PMC5968200; doi:10.3389/fmicb.2018.01023)

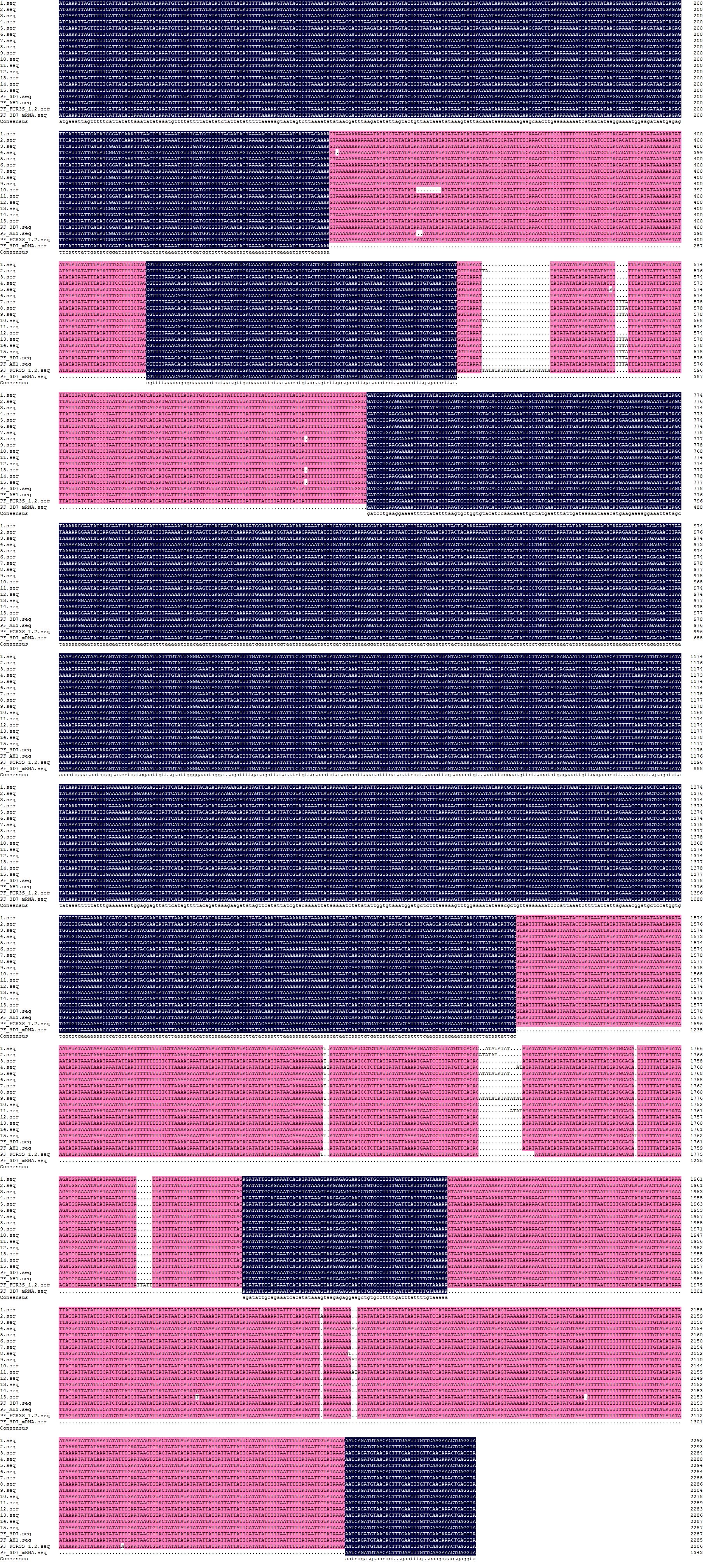

Supplement: FIGURE S1 — Sequence alignment analysis of the genes encoding TatD-like DNase in various P. falciparum strains and wild isolates. Numbers 1 to 15 represent the sequences amplified from wild type strains. PF_3D7, PF_AH1, PF_FCR3S represent the genomic sequences of the corresponding strain. PF_3D7_mRNA represents the mRNA sequence the P. falciparum 3D7 strain. The protein coding regions (dark blue) are completely conserved, whereas the differences only occurred in intron domains (white and pink). [file Image_1.JPEG]

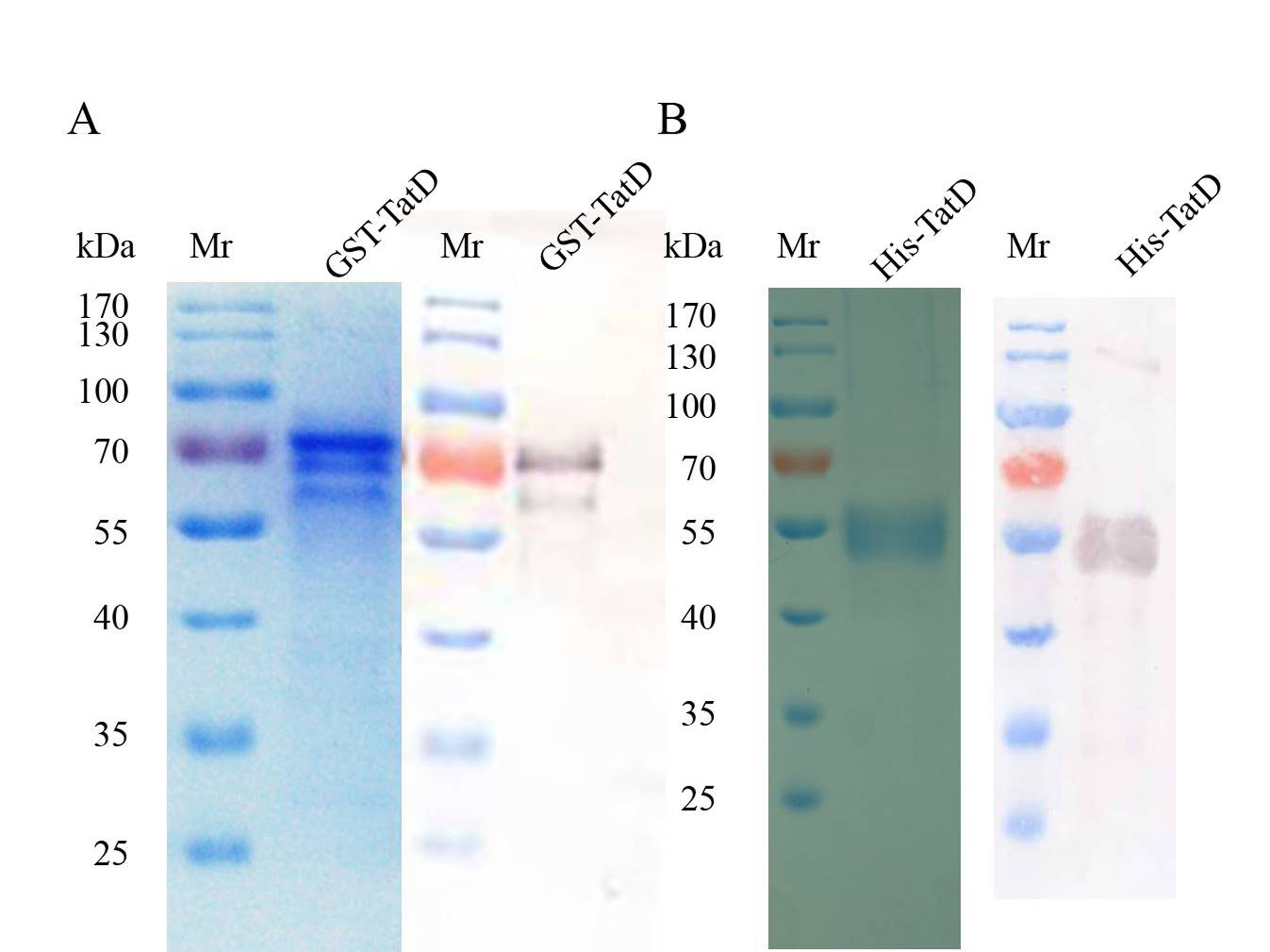

Supplement: FIGURE S2 — Purification and confirmation of His- and GST-tagged recombinant proteins. Quality of the purified GST-tagged TatD-like DNase (A), His-tagged TatD-like DNase (B). TatD-like DNase was determined by Coomassie stained SDS-PAGE gels and Western blots with tag-specific antibodies. [file Image_2.JPEG]
